# The population genetics of parasitic nematodes of wild animals

**DOI:** 10.1186/s13071-018-3137-5

**Published:** 2018-11-13

**Authors:** Rebecca Cole, Mark Viney

**Affiliations:** School of Biological Sciences, University of Bristol, Bristol, BS8 1TQ UK

**Keywords:** Helminths, Nematodes, Population genetics, Population genomics, Sequencing, Wild animals, Population structure, Conservation, Parasite ecology

## Abstract

Parasitic nematodes are highly diverse and common, infecting virtually all animal species, and the importance of their roles in natural ecosystems is increasingly becoming apparent. How genes flow within and among populations of these parasites - their population genetics - has profound implications for the epidemiology of host infection and disease, and for the response of parasite populations to selection pressures. The population genetics of nematode parasites of wild animals may have consequences for host conservation, or influence the risk of zoonotic disease. Host movement has long been recognised as an important determinant of parasitic nematode population genetic structure, and recent research has also highlighted the importance of nematode life histories, environmental conditions, and other aspects of host ecology. Commonly, factors influencing parasitic nematode population genetics have been studied in isolation, such that an integrated view of the drivers of population genetic structure of parasitic nematodes is still lacking. Here, we seek to provide a comprehensive, broad, and integrative picture of these factors in parasitic nematodes of wild animals that will be a useful resource for investigators studying non-model parasitic nematodes in natural ecosystems. Increasingly, new methods of analysing the population genetics of nematodes are becoming available, and we consider the opportunities that these afford in resolving hitherto inaccessible questions of the population genetics of these important animals.

## Background

Parasitic nematode infection is ubiquitous in wild animals and can profoundly alter the physiology, behaviour and reproductive success of hosts [1, 2], and as such parasitic nematodes play key roles in ecosystem functioning [3, 4]. However, parasitic nematodes can raise conservation concerns - invasive parasitic nematodes may threaten naïve, native hosts [5], while environmental changes may render hosts more susceptible to pre-existing parasitic nematode species, resulting in more severe disease [6]. Furthermore, parasitic nematodes that normally infect wild animals can spill-over into human and domestic animal populations, acting as new sources of disease [7, 8].

Population genetic structure - the distribution of genetic variation in time and space - affects how a species responds to selection pressures, and so shapes its evolution [9]. Studying the population genetics of parasites in wild animals has several benefits. First, it provides an insight into the parasite’s infection dynamics [9–11], with consequences for ecosystem functioning [3, 4]. Secondly, population genetic studies can reveal complexes of morphologically indistinguishable, but genetically very different, cryptic species, which are common among nematodes [12–14]. Thirdly, the patterning of population genetic structure of parasitic nematode species can inform on parasite phylogeography (see Table 1), and in so doing also clarify aspects of host phylogeography [15–19]. Finally, by studying the population genetics of parasitic nematodes in wild animals, ecological drivers of population genetic patterns in parasitic nematodes which may not be apparent in human- and livestock-infecting species can be identified.

**Table 1:** Glossary

Term	Definition
Census size (N)	The number of individuals in a population
Effective population size (Ne)	Effective population size (Ne): The number of individuals needed in an idealised population to explain the rate of change in allele frequency, or to explain the observed degree of inbreeding, observed in a real population [180]
Environmental DNA (eDNA)	DNA released by organisms into the environment [174]
Fixation	When only a single allele remains at a formerly-polymorphic locus
Gene flow	Movement of alleles among populations [181]. Tends to reduce genetic differentiation
Genetic drift	Stochastic change in allele frequencies within a population across generations [180]. Tends to increase genetic differentiation
Hardy-Weinberg equilibrium	The situation in which the number of heterozygotes observed matches the number of heterozygotes expected given the allele frequencies in the population [182]
Idealised population	A theoretical population in which each individual produces an infinite number of gametes, any gamete may fuse with any other of the opposite sex with equal probability, sex ratios are even, and there is no overlap in generations [180]
Infrapopulation	All the parasites of one species within one host individual [21]
Isozymes	Different, usually allelic, forms of an enzyme, which can be detected by differences in electrophoretic charge [183]
Linkage disequilibrium	The joint inheritance of particular alleles at different loci more often than would be expected by chance, usually due to their close physical proximity on a chromosome [184]
Phylogeography	Historical drivers of the current geographical distribution of a species [185]
Polymorphic	Sequence variation at a locus, classically with multiple alleles present at frequencies of 5% or greater [186]
Population (of a parasite)	A group of parasite infrapopulations that may exchange individuals freely [21]
Population bottleneck	Loss of a large, random portion of the population, resulting in reduced genetic diversity
Random amplified polymorphic DNA (RAPD)	DNA fragments amplified by a defined set of arbitrary PCR primers, polymorphic due to inter-individual differences in primer binding sites [187]
Ribosomal internal transcribed spacers (ITS)	Putatively non-functional stretches of DNA between the ribosomal RNA-encoding genes in eukaryotes. [188]. The first and second ITS are denoted ITS1 and ITS2 respectively
Selective sweep	An increase in the frequency of a set of alleles owing to their genetic linkage to an allele undergoing positive selection [189]

The population genetics of metazoan parasites, including nematodes, has been reviewed extensively [9–11, 20–25], but much of this information is based on species that infect people and livestock. It is not clear how applicable these findings are to nematodes whose hosts establish their populations naturally. Parasites’ life histories, their hosts’ life histories, and the extra-host environment will all contribute to parasites’ population genetic structure [10, 11, 22, 23, 25], but little is known about the relative importance of these factors in natural ecosystems, and how they interact.

Here, we provide a resource that collates what is known about the population genetics of parasitic nematodes in natural ecosystems, that we envisage will be useful to researchers investigating the important, but little-understood, roles that parasitic nematodes play in such ecosystems. To this end, we comprehensively review the population genetics of parasitic nematodes in wild animals, including every relevant study of which we are aware. We take wild animals to be ones that establish populations without direct human management, even if they live commensally in human settlements (such as cities), or if their habitat is undergoing anthropogenic changes, since these host populations may nonetheless influence the population genetics of their parasitic nematodes in ways that hosts whose populations are managed by humans cannot. We consider the parasitic nematodes of terrestrial vertebrates, marine vertebrates, and arthropods, and draw together the evidence to present a synthesis on the factors determining the population genetics of parasitic nematodes in wild animals. We examine the population genetics of parasitic nematodes in wild animals that have undergone recent habitat change, asking how parasite populations respond to anthropogenic influences on natural systems. Finally, we assess future prospects in the study of parasitic nematode population genetics, discussing opportunities provided by high-throughput DNA-sequencing-based methods, and highlighting the importance of including extra-host stages in population genetics studies.

## Parasitic nematodes of wild terrestrial vertebrates

### Parasitic nematodes of marsupials

Commonly, population genetic studies of the parasitic nematodes that infect macropod marsupials reveal that what was previously classified as a single nematode species with a broad host range is actually a complex of cryptic species, each with a narrow host range (Table 2). This highlights the ability of population genetic techniques to detect species complexes. In some cases, these studies also show whether, and if so, how, populations within these cryptic species are geographically structured. For example, geographical structuring of genetic diversity was not seen in populations of *Globocephaloides trifidospicularis* [26], nor in several species within the *Hypodontus macropi* complex [27], nor in *Rugopharynx australis* from *Macropus robustus* and *M. rufus* [28], nor in several *Capillaria* species [29], but was observed in populations of *H. macropi* from subspecies of *Macropus robustus*, *H. macropi* from subspecies of *Macropus rufogriseus* [27] and* Labiosimplex* *australis* from *M. rufogriseus* [30]. In some cases, studies have failed to detect any genetic variation within parasitic nematode species at all, such as in *H. macropi* from *Petrogale persephone* [27], *Globocephaloides affinis* from *Macropus dorsalis* [26], and several other *Cloacina* species [31], but this may be due to the very low numbers of hosts and parasites studied (Table 2).

**Table 2:** Species complexes within strongylid nematode parasites of marsupials

Nominal species	Host(s) sampled	Genetic marker(s)	Remarks	Reference
*Globocephaloides trifidospicularis*	*Macropus rufogriseus*; *M. giganteus* (host numbers not given)	24 isozymes (see Table 1)	Two host-specific species, with 4 fixed differences. ITS1 and ITS2 sequencing later failed to detect these species [26]	[190]
*Hypodontus macropi*	3 *Macropus agilis*; 4 *M. dorsalis*; 1 *M. rufogriseus rufogriseus*; 1 *M. rufogriseus banksianus*; 2 *M. robustus robustus*; 1 *M. robustus erubescens*; 2 *M. rufus*; 3 *M. fuliginosus*; 2 *Wallabia bicolor*; 2 *Thylogale billardierii*	28 isozymes	Six species detected, with fixed differences at 20–50% of loci. One in *M. r. erubescens*, *M. r. robustus*, *M. rufus* and *M. fuliginosus*, one in *M. r. banksianus* and *M. r. rufogriseus*, and one in each other host. Species status of nematodes in *M. r. robustus*, *T. billardierii* and *M. bicolor* later supported in [191]	[27]
*Rugopharynx omega*	2 *Macropus rufogriseus*; 1 *Thylogale stigmatica*	23 isozymes	*R. omega* from each host species had fixed differences at 10 loci. *R. omega* from *T. stigmatica* named *Rugopharynx sigma*	[192]
*Paramacropostrongylus typicus*	4 *Macropus fuliginosus*; 2 *M. giganteus*	37 isozymes	*P. typicus* from each host species had fixed differences at 10 loci. *P. typicus* from *M. giganteus* named *Paramacropostrongylus iugalis*. Hybridisation later detected [193]	[194]
*Macropostrongylus baylisi*	2 *Macropus giganteus*; 15 *M. robustus robustus*; 4 *M. r. erubescens*; 15 *M. parryi*	27 isozymes	*M. baylisi* from *M. giganteus* had fixed differences at 9 loci compared with *M. baylisi* from other hosts examined	[195]
*Rugopharynx zeta*	1 *Petrogale assimilis*; 2 *Macropus dorsalis*	21 isozymes	*R. zeta* from *P. assimilis* had 10 fixed differences when compared with *R. zeta* from *M. dorsalis*. *M. dorsalis* parasite named *Rugopharynx mawsonae*	[196]
*Rugopharynx australis*	1 *Macropus eugenii*; 5 *M. fuliginosus*; 3 *M. giganteus*; 2 *M. robustus*, 2 *M. rufogriseus*, 3 *M. rufus*; 2 *Thylogale billardierii*; 1 *Wallabia bicolor*	17 isozymes	Six species found, with fixed differences at up to 50% of loci. One in *M. robustus* and *M. rufus*, one in *M. giganteus* and *M. fuliginosus*, 2 in *M. rufogriseus* and one each in the remaining hosts	[28]
*Labiostrongylus uncinatus*	1 *Macropus dorsalis*; 2 *M. parryi*	17 isozymes	*L. uncinatus* from each host species had fixed differences at 13 loci. *L. uncinatus* from *M. parryi* named *Labiostrongylus contiguus*	[197]
*Labiostrongylus bancrofti*	1 *Macropus dorsalis*; 1 *M. parryi*	18 isozymes	*L. bancrofti* from each host species had fixed differences at 15 loci. *L. bancrofti* from *M. parryi* named *Labiostrongylus turnbulli*	[198]
*Zoniolaimus mawsonae*	9 *Macropus rufus*	ITS2	Two sympatric taxa had fixed differences at 3 out of 230 nucleotides. One named *Zoniolaimus latebrosus*	[199]
*Papillostrongylus labiatus*	1 *Macropus dorsalis*; 1 *M. rufus*	ITS2	Two taxa, one in *M. dorsalis* and one in *M. rufus,* had fixed differences at 40 out of 240 nucleotides. Taxon in *M. rufus* named *Papillostrongylus barbatus*	[200]
*Cloacina ernabella*	1 *Petrogale purpureicollus*	ITS1 and ITS2	Geographically isolated taxa had fixed differences at 13 of 606 nucletides	[31]
*Cloacina caenis*	1 *Petrogale assimilis*; 1 *P. herberti*; 1 *P. inornata*; 1 *P. mareeba*; 1 *P. purpureicollis*	ITS1 and ITS2	Four taxa identifiable by 1–4 fixed differences. One each from *P. purpureicollis*, *P. herberti* and *P. mareeba*, and one in *P. assimilis* and *P. inornata*	[31]
*Cloacina pearsoni*	1 *Petrogale assimilis*; 1 *P. herberti*; 1 *P. inornata*; 1 *P. mareeba*; 1 *P. purpureicollis*	ITS1 and ITS2	Five taxa, identifiable by 2–9 fixed differences. One taxon in each host species	[31]
*Cloacina robertsi*	1 *Petrogale assimilis*; 1 *P. herberti*; 1 *P. inornata*; 1 *P. mareeba*; 1 *P. purpureicollis*; 1 *P. persephone*	ITS1 and ITS2	Two taxa, identifiable by 10 fixed differences. One taxon in *P. persephone*, the other in all other host species.	[31]
*Globocephaloides macropodis*	4 *Macropus dorsalis*; 1 *M. agilis*	ITS1 and ITS2	*G. macropodis* from each host species had fixed differences at 5.2% and 7.1% of nucleotides in ITS1 and ITS2, respectively	[26]

Why do some of these parasite species have genetic population structuring while others do not? There was genetic differentiation between *L. australis* collected from Tasmania and Kangaroo Island, and between both of these populations and mainland Australia [30]. Similarly, Tasmanian populations of *H. macropi* in *M. rufogriseus* showed genetic differentiation from that on mainland Australia [27]. This suggests that marsupial hosts are ineffective at mediating nematode transmission across open water. However, a cryptic species of *R. australis* in *M. giganteus* and *M. fuliginosus* did not show population differentiation despite being sampled from both Kangaroo Island and mainland Australia [28], nor did *G. trifidospicularis* sampled from multiple macropod species [26]. This may indicate ongoing gene flow in these species between Kangaroo Island and the mainland, or alternatively, that one or both of these species only recently arrived on Kangaroo Island, and the island parasite populations have yet to differentiate detectably from their mainland counterparts.

Genetic differentiation is rarely detected within marsupial-infecting nematode species collected from only mainland Australia; it is apparently absent in three cryptic species of *H. macropi* in *Macropus dorsalis*, *M. agilis* and *Wallabia bicolor*, respectively [27], as well as in a single species within *R. australis* collected from *M. robustus* and *M. rufus* [28]. However, Queensland populations of a cryptic species of *H. macropi* recovered from *M. robustus* were found to be differentiated from those in South Australia [27]. This may simply reflect the size of the study area; sampling locations of *H. macropi* in *M. robustus* were over 1500 km apart, compared with up to 800 km in other species. Naturally, as distances between populations increase, host-mediated transmission between the populations becomes rarer, though the exact distances over which host-mediated transmission becomes inefficient will likely vary with the vagility of host species. It should be noted that in many cases these studies involved low numbers of hosts and parasites (Table 2), and so population genetic insights gleaned from them should be treated with caution. Further work using much larger sample sizes is needed to confirm the patterns of population genetic structure in the parasitic nematodes of marsupials that many existing studies suggest.

### Parasitic nematodes of terrestrial carnivores: *Trichinella*

*Trichinella* spp. have broad geographical ranges and broad host species ranges, with each species parasitizing a variety of carnivorous vertebrates. *Trichinella* spp. share certain population genetic characteristics, such as differentiation among infrapopulations [32, 33] (see Table 1) and low intra-specific genetic diversity [32–39]. *Trichinella* spp. tend to show population genetic structuring among continents; for example, genetic differentiation was found among populations of *T. pseudospiralis* from Australia, North America, Europe and Asia [38, 40], and among *T. spiralis* populations from Asia and Europe [33]. *Trichinella nelsoni* from Kenya and Tanzania was differentiated from that in South Africa [37], but this parasite was recovered from only one host individual in each country, meaning that infrapopulation differentiation was not accounted for.

The apparent lack of population genetic differentiation in *Trichinella* spp. within continents matches observations made of other nematodes that parasitise large carnivorous mammals [41*–*43], and likely arises from long-distance dispersal of hosts, which promotes high parasite gene flow. For *Trichinella* spp., gene flow may also be promoted by smaller hosts (such as rats and foxes), facilitating parasite gene flow among otherwise discontiguous populations of very mobile hosts.

But what factors drive the among-host differentiation and low intra-specific diversity seen in *Trichinella* spp.? *Trichinella* transmission stages remain in the muscle of their parents’ host and infection of a new host occurs by predation [44]. This life-cycle may lead to clumped transmission of siblings, potentially resulting in differentiation among infrapopulations and promoting inbreeding. Inbreeding tends to reduce effective population size (Ne, see Table 1), leading to stronger genetic drift (see Table 1), meaning that alleles are more readily lost from the population, reducing genetic diversity. Indeed, clumped transmission of related parasites has previously been suggested to promote low genetic variation and genetic differentiation among hosts in diverse parasite taxa (reviewed in [9, 10, 21]).

In summary, the population genetics of *Trichinella* spp. appears to be driven by (i) highly mobile hosts and a broad host range, promoting gene flow, and (ii) its life-cycle, promoting clumped transmission of sibling parasites and so lowering Ne.

### Parasitic nematodes of rodents

The limited dispersal of wild rodent individuals [45–48] might be expected to limit gene flow in their nematode parasites, resulting in genetic structure over small geographical scales. Accordingly, populations of *Heligmosomoides polygyrus*, a parasite of the European woodmouse (*Apodemus sylvaticus*), show extensive population genetic structure across the host species’ range [15, 49–51], with *H. polygyrus* populations being more strongly differentiated than those of *A. sylvaticus* according to mitochondrial sequence analysis of both species [15]. *Heligmosomoides polygyrus* has a faster mitochondrial mutation rate and generation time than its host [15, 52], likely meaning that mitochondrial genetic drift is faster in *H. polygyrus* compared with *A. sylvaticus* and so contributing to the comparatively stronger population genetic structure of *H. polygyrus*.

*Trichuris muris* infects rats and mice (including *A. sylvaticus*), while *T. arvicolae* infects arvicoline rodents (lemmings and voles), and both *Trichuris* spp. are found throughout Europe. Like *H. polygyrus*, *T. muris* and *T. arvicolae* both show extensive population genetic structure across their geographical range, as determined by analysis of both mitochondrial and nuclear loci [53–55]. Indeed, broadly similar patterns of population genetic structure were observed in *H. polygyrus* and both *Trichuris* spp., with a delineation between eastern and western populations, and greater diversity in southern populations, compared with northern. These patterns may reflect range expansion of the rodent hosts from southern refugia during the last ice age, at least 12,000 years ago [15, 53–55]. Stronger population genetic structuring and a smaller geographical range was observed in *H. polygyrus* than in *Trichuris* spp. [15, 53–55]. This may be partly due to faster genetic drift in *H. polygyrus* than in *Trichuris* spp., arising from a shorter generation time in the former compared with the latter (~14 days and 50–60 days, respectively) [56, 57], or may reflect the broader host range of *Trichuris* spp. A broader host range may allow a parasite to occupy and traverse a wider range of environments, potentially increasing gene flow rates and slowing genetic drift [23].

The population genetics of *Angiostrongylus cantonensis* and *A. malaysiensis*, parasites of rodents of the family Muridae [58], has been extensively studied, with genetic structure detected on both small and large geographical scales [59–69]. However, interpretation of these findings is made difficult by the recent discovery of at least two sympatric cryptic species within *A. cantonensis*, one of which may be conspecific with individuals identified as *A. malaysiensis* [70, 71]. It is now not clear whether the population genetic structure detected previously really represents the geographical distribution of genetic variants in a single species, or rather results from the accidental sampling of multiple reproductively isolated species. Nevertheless, if this cryptic speciation is taken into account, the population genetic data of *A. cantonensis* (*sensu stricto*) and *A. malaysiensis* can be examined. In both species, population genetic structure was detected among provinces in Thailand [70]. It is likely that limited vagility in both the definitive rodent host and the intermediate snail host limits gene flow in *Angiostrongylus* spp. over large distances.

Other studies have investigated parasitic nematode population genetic structure at finer geographical scales. For example, *Neoheligmonella granjoni*, a parasite of multimammate mice (*Mastomys* spp.), was sampled from *M. natalensis* and *M. erythroleucus* within a 70 km^2^ rural area of Senegal, and genotyped at 10 microsatellite loci [72]. These data revealed an absence of genetic population structure, with alleles being distributed homogenously among sampling sites. This may be due to the relatively high dispersal of *M. erythroleucus*, which will promote gene flow across the study area, including among populations of the much more sedentary host, *M. natalensis* [72, 73]. A lack of population genetic structure over fine geographical scales (e.g. within a 100 km^2^ area) was also seen in *Longistriata caudabullata*, a nematode that parasitises *Blarina* sp. shrews [74], showing that while parasitic nematodes of rodents and other small mammals show strong genetic structure at broad geographical scales, at finer scales, gene flow can be sufficient to genetically homogenise populations.

*Strongyloides ratti* is a parasite of brown rats, *Rattus norvegicus*, and shows little genetic differentiation among UK sampling sites ~20–250 km apart [75]. This may indicate that *R. norvegicus* dispersal is sufficient to genetically homogenise the *S. ratti* population at these scales. While *S. ratti* did not show genetic differentiation among host populations, there was differentiation among infrapopulations [75]. *Strongyloides* spp. are unusual because the parasitic adults reproduce clonally, so that all of a single parasite’s offspring are genetically identical [76], and along with clumped transmission of clonal siblings [77], this may lead to the observed among-host differentiation.

Analysis of *Syphacia stroma* and *H. polygyrus* from the same *A. sylvaticus* host individuals showed that *S. stroma* has substantially lower genetic diversity and higher population differentiation than *H. polygyrus* [78]. *Syphacia stroma* has haplodiploid sex determination, in which haploid males develop from unfertilised eggs produced by diploid females, while in *H. polygyrus* males and females are both produced sexually. Mating system is recognised as an important factor in parasite population genetics [10, 11, 23], and so the different mating systems of *H. polygyrus* and *S. stroma* may explain their different population genetic structures. Haplodiploidy lowers Ne by reducing the number of individuals contributing to the next generation (because males are produced from the mother’s genetic material only), and this may lead to greater genetic drift in *S. stroma* compared with *H. polygyrus*. *Syphacia stroma* and *H. polygyrus* have broadly similar generation times [79] and share a host, so their different mating systems emerge as likely important factors behind their different population genetics. Aspects of life history such as mating system have not been extensively studied in parasitic nematodes of wild animals, and further studies in this area may contribute to our understanding of population genetics in other parasitic nematode species.

In summary, the population genetics of parasitic nematodes in wild rodents appears to be defined largely by hosts’ low dispersal ranges. However, different patterns of population genetic structure among parasite species sharing a host species suggest that parasite mating system and generation time are also influential.

### Parasitic nematodes of ungulates

Ungulate (hoofed mammal) individuals travel over much greater distances than rodents, and so may facilitate comparatively greater gene flow of their parasitic nematodes. *Ostertagia gruehneri* and *Marshallagia marshalli*, both parasites of reindeer (*Rangifer tarandus*), show a lack of population genetic structuring [80, 81], a pattern similar to that of *Teladorsagia boreoarcticus* in muskoxen (*Ovibos moschatus*) [82]. In contrast, *Mazamastrongylus odocoilei*, a parasite of white-tailed deer (*Odocoileus virginianus*), showed genetically structured populations [83]. This difference may reflect the more rapid evolution of mtDNA, used to study *M. odocoilei*, compared with the internal transcribed spacer (ITS, see Table 1) sequences used for *O. gruehneri* and *M. marshalli*. However, species-specific differences in host ecology may also contribute to the different patterns of population genetic structure seen among *O. gruehneri*, *M. marshalli* and *M. odocoilei* - reindeer have large home ranges and are partially migratory [84], and so may provide more opportunities for gene flow in their parasitic nematodes compared with the more sedentary white-tailed deer [85].

*Dictyocaulus eckerti* is a parasite of several species of deer (*Cervus* spp. and *Dama* spp.). Analysis of mitochondrial sequence data found weak genetic structuring in *D. eckerti* [86], while *D. capreolus* (specific to roe deer, *Capreolus capreolus*), had comparatively lower genetic diversity and more strongly genetically structured populations when sampled sympatrically [86]. *Dictyocaulus capreolus* is susceptible to population bottlenecks if roe deer numbers fall, whereas *D. eckerti* can weather a crash in the population of any one host species by persisting in other host species, and thereby maintain a high census size (N, see Table 1). High genetic diversity and a genetically unstructured populations is also observed in *Trichostrongylus axei*, which parasitises diverse wild ungulate species [87], suggesting an association between these population genetic traits and broad host range. Differences in host behaviour may also contribute to the differences in the population genetics of *D. eckerti* and *D. capreolus*; specifically, *D. eckerti* may have higher gene flow than *D. capreolus* because of the territorial nature of roe deer, which limits the geographical distances they cover.

### Parasitic nematodes of reptiles and amphibians

*Spauligodon anolis* infects anole lizards (*Anolis* spp.), while *Parapharyngodon cubensis* is a species complex (*P. cubensis* A, *P. cubensis* B and *P. cubensis* C) together infecting a broad range of lizards and snakes. Study of the population genetics of these nematodes, sampled from various Caribbean *Anolis* spp. hosts, found that genetic diversity was partitioned both among and within islands [88]. However, *S. anolis* populations were more strongly genetically differentiated than populations of *P. cubensis* A or *P. cubensis* B, likely because *S. anolis* has a narrow host species range made up of poor dispersers [89], while the species of the *P. cubensis* complex each make use of a wider range of hosts, among which may be more mobile host species [88]. In contrast, cryptic species within *Spauligodon atlanticus*, parasites of *Gallotia* spp. lizards, all showed strong genetic structuring within and among islands of the Canary Isles, despite differing in the extent of their host range [90]. This may be because the geographical ranges of the host species of *S. atlanticus* do not overlap, precluding nematode gene flow between them.

Population genetic analysis of *Rhabdias ranae*, a parasite of the northern leopard frog (*Lithobates pipiens*), revealed low microsatellite heterozygosity, differentiation among infrapopulations and population genetic structure at a very fine scale, with differentiation emerging among ponds less than 1 km apart [91]. *Rhabdias ranae* is a specific parasite of *L. pipiens* and lacks an intermediate host, so its dispersal is likely mediated almost entirely through *L. pipiens* movement. Hence, sibling extra-host stages are likely to remain clumped in the environment and infect a new host together, explaining infrapopulation differentiation. If *L. pipiens* habitually visit the same locations (e.g. show fidelity to a particular breeding pond), then they may even be re-infected with the offspring of their own parasites [91]. Such a habit might also explain differentiation among ponds, if the same cohort of frogs routinely utilise a particular pond [91]. Low heterozygosity is likely a product of inbreeding, arising from the life-cycle of *R. ranae*, which includes a self-fertilising hermaphroditic stage.

### Parasitic nematodes of terrestrial birds: *Trichostrongylus tenuis*

*Trichostrongylus tenuis* is a strongylid parasite of galliform and anseriform birds, and is particularly prevalent in red grouse (*Lagopus lagopus scotica*). Population genetic analysis of *T. tenuis* in UK red grouse revealed high microsatellite diversity, and a lack of population genetic structure among host individuals and among geographically separated populations [92, 93]. This lack of population genetic structure is likely due to the very high prevalence and infection intensity of this parasite [94], presumably leading to a high Ne, so rendering genetic drift very slow. Population genetics can also be used to study parasite dispersal. For example, a lack of genetic differentiation between *T. tenuis* in a goose in Iceland and those in UK grouse suggests long distance *T. tenuis* gene flow. Some waterfowl species, such as the pink-footed goose (*Anser brachyrhynchus*), migrate between the UK and Iceland [95], presenting a possible avenue for *T. tenuis* gene flow between these countries.

## Parasitic nematodes of aquatic vertebrates

### Parasitic nematodes of marine mammals and birds

Most nematode parasites of marine mammals and birds are trophically transmitted among intermediate hosts before reaching the definitive host [96]. As hosts of each trophic level are likely to consume multiple infected hosts in the lower trophic levels, hosts will accumulate parasites from a variety of sources, and so definitive host individuals will probably sample widely from the parasite population. This may lead to genetically diverse parasite infrapopulations that obviate inbreeding and promote high Ne [11]. Because many marine fish, mammal and bird hosts travel large distances [97, 98], gene flow of their parasitic nematode populations is expected to be high, suggesting that these nematode populations will show little genetic structuring.

Many parasitic nematodes infecting marine vertebrates do indeed show little population genetic structure. *Anisakis simplex* is a complex of several cryptic species with different geographical and definitive host ranges [99–101]. Population genetic structure has rarely been observed within species of the *A. simplex* complex (Table 3), and it is likely that earlier reports of extensive genetic structure in *A. simplex* [102] resulted from inadvertent sampling of multiple species. A similar lack of population genetic structure has been observed in a variety of other nematodes with similar life histories, including other *Anisakis* spp. in pinnipeds and cetaceans, *Contracaecum* spp. from a variety of birds and mammals, and *Pseudoterranova* spp. from pinnipeds (Table 3). However, *Anisakis simplex* C may be an exception, with one study detecting genetic differentiation between northern and southern hemisphere populations [99]. Intra-taxon genetic diversity of parasitic nematodes of marine mammals in the southern hemisphere is generally greater than in the northern hemisphere, perhaps due to comparatively lower habitat disturbance (e.g. fishing, pollution) in the southern hemisphere [103].

**Table 3:** Parasitic nematodes of marine mammals and birds that do not show population genetic structure

Species	Sampled non-definitive hosts	Sampled definitive hosts	Regions sampled	Genetic marker(s)	Reference
*Anisakis pegreffii*	Squid (*Todarodes* spp., *Tadaropsis eblanae*), teleosts (families Bramidae, Carangidae, Clupeidae, Congridae, Emmelycthidae, Engraulidae, Gadidae, Gempylidae, Lophiidae, Merlucciidae, Myctophidae, Ophidiidae, Scombridae, Scorpaenidae, Sparidae, Trachichtyidae, Trichiuridae, Xiphiidae)	Cuvier’s beaked whale (*Ziphius cavirostris*), sperm whale (*Physeter microcephalus*), delphinid dolphins	Mediterranean, North-West Atlantic, Pacific off Falkland Islands, Madeira, New Zealand and South Africa	32 isozymes, ITS, mtDNA, RAPD	[99–101, 201–206]
*Anisakis simplex* (*sensu stricto*)	Squid (*Tadaropsis eblanae*, *Todarodes sagittus*, *Illex coindettii*), teleosts (families Carangidae, Clupeidae, Engraulidae, Gadidae, Merlucciidae, Pleuronectidae, Salmonidae, Scomberesocidae, Scombridae, Trichiuridae)	Beluga whale (*Delphinapterus leucas*), harbour porpoise (*Phocoena phocoena*), longfinned pilot whale (*Globicephala melas*), delphinid dolphins	North Sea, Norwegian Sea, Mediterranean Sea, Baltic Sea, Atlantic, North-West Pacific, off Japan and Madeira	32 isozymes, ITS1, 5.8S, ITS2, mtDNA, RAPD	[99–101, 202–209]
*Anisakis simplex* C	Teleosts (families Gadidae, Gempylidae, Myctophidae, Trachichtyidae)	Strap-toothed whale (*Mesoplodon layardii*), dolphins (*Pseudorca crassidens*, *Globicephala melas*)	Atlantic, Tasman Sea, North-West Pacific, off South Africa and New Zealand	24 isozymes, ITS1, 5.8S, ITS2, mtDNA, RAPD	[100, 101, 206]
*Anisakis paggiae*		Dolphins (*Delphinus delphis*, *Globicephala melas*, *Tursiops truncatus*), pygmy sperm whales (*Kogia breviceps*, *K. sima*)	Atlantic off Spain, off South Africa and USA	19 isozymes	[100]
*Anisakis brevispiculata*		Pygmy sperm whale (*Kogia breviceps*)	Off Spain and South Africa	22 isozymes	[210]
*Anisakis physeteris*	Teleosts (families Carangidae, Gadidae, Scombridae)	Sperm whale (*Physeter microcephalus*)	Mediterranean Sea, off Madeira	21 isozymes, ITS1, 5.8S, ITS2	[101, 210, 211]
*Anisakis typica*	Teleosts (families Carangidae, Coryphaenidae, Merlucciidae, Trichiuridae, Scombridae)	Delphinid dolphins, harbour porpoise (*Phocoena phocoena*) short-finned pilot whale (*Globicephala macrorhynchus*), La Plata dolphin (*Pontoporia blainvillei*)	Off Florida, Madeira, Somalia and Brazil, Mediterranean Sea	20 isozymes, ITS1, 5.8S, ITS2	[99, 101, 202]
*Anisakis ziphidarum*	Teleosts (families Carangidae, Scombridae, Trichiuridae)	Strap-toothed whale (*Mesoplodon layardii*) and Cuvier’s beaked whale (*Ziphius cavirostris*)	Off Madeira	22 isozymes, ITS1, 5.8S, ITS2	[101, 212]
*Anisakis nascettii*	Black scabbardfish (*Aphanopus carbo*) and chub mackerel (*Scomber japonicus*)	Beaked whales (*Mesoplodon* spp.)	Off Madeira, Spain, New Zealand and South Africa	ITS1, 5.8S, ITS2 mtDNA	[101, 213–215]
*Pseudoterranova decipiens* A	Teleosts (families Gadidae, Pleuronectidae, Scopthalmidae)	Grey seal (*Halichoerus grypus*), harbour seal (*Phoca vitulina*)	North-East Atlantic	9 isozymes	[216]
*Pseudoterranova decipiens* B	Teleosts (Cottidae, Gadidae, Lotidae, Pleuronectidae)	Grey seal (*Halichoerus grypus*), harbour seal (*Phoca vitulina*) hooded seal (*Cystophora cristata*)	North Atlantic	9 isozymes	[216]
*Pseudoterranova decipiens* C	American plaice (*Hippoglossoides platessoides*)	Bearded seal (*Erignathus barbatus*)	North Atlantic	9 isozymes	[216]
*Contracaecum* spp.	Topminnows (*Poeciliopsis* spp.) and the cichlid *Cichlasoma beani*		Off Arroyo Aguajita (Mexico)	17 isozymes	[217]
*Contracaecum osculatum* A		Bearded seal (*Erignathus barbatus*), grey seal (*Halichoerus grypus*),	North Atlantic	17 isozymes	[218]
*Contracaecum osculatum* B		Bearded seal (*Erignathus barbatus*), grey seal (*Halichoerus grypus*), harp seal (*Phoca groenlandica*), harbour seal (*Phoca vitulina*)	North Atlantic	17 isozymes	[218]
*Contracaecum osculatum* C		Grey seal (*Halichoerus grypus*)	North-East Atlantic	17 isozymes	[218]
*Contracaecum osculatum* D	Teleosts (families Channichthyidae, Nototheniidae)	Weddel seal (*Leptonychotes weddellii*)	Antarctic Southern Ocean	24 isozymes, mtDNA	[219, 220]
*Contracaecum osculatum* E	Teleosts (families Channichthyidae, Nototheniidae)	Weddel seal (*Leptonychotes weddellii*)	Antarctic Southern Ocean	24 isozymes, mtDNA	[219, 220]
*Contracaecum radiatum*	Crocodile icefishes (*Chionodraco hamatus* and *Cryodraco antarcticus*)	Weddel seal (*Leptonychotes weddellii*)	Antarctic Southern Ocean	24 isozymes	[221]
*Contracaecum ogmorhini*		Fur seals (*Arctocephalus* spp.)	Off Australia, Argentina and South Africa	18 isozymes, ITS1, ITS2, mtDNA	[222, 223]
*Contracaecum margolisi*		Caliphornia seal lion (*Zalophus californianus*)	Pacific off Canada	18 isozymes, ITS1, ITS2, mtDNA	[222, 223]
*Contracaecum rudolphii* A		Cormorants (*Phalacrocorax* spp.)	Atlantic off Spain, off Poland and Italy	20 isozymes, ITS1, ITS2, mtDNA	[224, 225]
*Contracaecum rudolphii* B		Continental great cormorant (*Phalacrocorax carbo sinensis*)	Off Italy and Poland	20 isozymes, ITS1, ITS2, mtDNA	[224, 225]
*Contracaecum septentrionale*		Continental great cormorant (*Phalacrocorax carbo sinensis*)	Off Iceland and Norway	20 isozymes, mtDNA	[225]

*Uncinaria sanguinis*, a parasite of the Australian sea lion (*Neophoca cinerea*), requires adult female hosts on breeding beaches to complete its life-cycle [104], and female hosts always return to the beach they were born on to breed [105]. One might therefore expect *U. sanguinis* to show genetic differentiation among host breeding beaches, but in fact no population genetic structure was observed in *U. sanguinis* at all [106], and a similar situation is seen in *Uncinaria lucasi* infecting northern fur seals (*Callorhinus ursinus*) [107]. This may indicate that the life-cycle of *Uncinaria* spp. is not fully understood and that transmission also occurs in other ways - male sea lions do move among breeding beaches [105], so transmission involving males could homogenise parasite population genetic structure. Hence, population genetic studies can suggest hypotheses about transmission cycles of parasitic nematodes that might otherwise be unexpected.

### Parasitic nematodes of fish

Some fish species travel around the globe, while others spend their whole lives in a small home patch, and this diversity in movement behaviour is likely to affect the population genetics of their parasitic nematodes. *Hysterothylacium aduncum* is a poorly-defined nematode species complex that infects a broad range of marine fish species [108]. ITS sequence data of *H. aduncum* from sprats (*Sprattus sprattus*) in western Europe showed two genetically distinct populations: one in the English Channel and the Bay of Biscay, and one in the Mediterranean and North Sea [109]. The geographical separation of the Mediterranean and North Sea (and that they are separated by the English Channel and Bay of Biscay), makes this parasite population genetic structure peculiar, and it contrasts with the population genetics of the sprats [110]. Potentially, another host species may be responsible for genetically homogenising the *H. aduncum* populations in the Mediterranean and North Sea *via* migration, and sampling of *H. aduncum* from other hosts is needed to test this hypothesis. In contrast with *H. aduncum*, there was no genetic structure in *Hysterothylacium fabri* within the Mediterranean, when considering either geography or host fish species [111].

Parasites of fish in discontiguous water bodies can become genetically distinct. For example, splitfin fishes (several genera within the Goodeidae) live in a series of unconnected lakes in Mexico, and their parasite, *Rhabdochona lichtenfelsi*, shows strong genetic differentiation among lakes, with the degree of differentiation correlating with the time since the lakes became separated [112]. In contrast, populations of the yellowhead catfish (*Pelteobagrus fulvidraco*) parasite *Procamallanus fulvidraconis* in isolated lakes were not significantly genetically different from each other [113]. These lakes were connected until the 1950s [114], so there may have been insufficient time for the parasite populations to diverge genetically. *Camallanus cotti* parasitizes a variety of freshwater fish species, and it showed no population genetic structure among the Yangtze and Minjiang river systems (geographically close and possibly occasionally connected by flood water), though populations from the Pearl River were distinct [115].

Collectively, studies of the population genetics of parasitic nematodes in aquatic environments reveal that their population genetic structures emerge at the scale over which hosts move, with genetically unstructured populations being common. Population genetic structure can emerge in these parasites when populations are restricted to isolated water bodies, or where host movement is constrained.

## Nematode parasites of arthropods

Virtually all invertebrate taxa are infected by parasitic nematodes, but the life histories of these nematodes are often poorly understood, and their population genetics barely explored. The insect parasite *Heterorhabditis marelatus* has a low level of mitochondrial genetic diversity and shows extreme population genetic structuring among sample sites (7 to 890 km apart) [116]. This may arise from very low gene flow in *H. marelatus*, since the extreme pathogenicity of *H. marelatus* kills hosts before they can carry their parasites far, preventing host movement from contributing significantly to parasite dispersal [116]. The life-cycle of *H. marelatus* may also contribute to its strong population genetic structure; *Heterorhabditis* spp. infections are initiated by juveniles which, upon maturation into hermaphrodites, self-fertilise to produce males and further hermaphrodites that continue to reproduce on the host’s cadaver [117]. This life-cycle promotes frequent founder effects (when an infective juvenile invades a host) and inbreeding (self-fertilisation and sib-mating), together driving low Ne. Low genetic diversity and population genetic structure was also observed in *Strelkovimermis spiculatus*, sampled from mosquito larvae (*Aedes* spp. and *Culex pipiens*), with genetic differentiation observed among ponds ~7 to 14 km apart [118]. In contrast, no population genetic structure was observed in *Isomermis lairdi*, a parasite of larval blackflies (*Simulium* spp.), when sampled from three rivers and multiple host species [119]. That the rivers were connected likely facilitates gene flow of *I. lairdi*, resulting in less structured populations compared with *S. spiculatus*.

*Thaumamermis zealandica*, a parasite of the sandhopper (*Bellorchestia quoyana*, a beach-dwelling amphipod), showed a complete absence of genetic diversity in three mitochondrial protein-coding genes when sampled from numerous hosts along an ~580 km stretch of New Zealand coast [120]. This could result from (i) panmixia among sampling sites and an extremely low Ne in the entire population, such that genetic drift affects *T. zealandica* at all sampling sites as a single population; (ii) a very recent population bottleneck; (iii) an extremely low mitochondrial mutation rate; or (iv) a combination of these factors [120]. Extremely low genetic variation in mitochondrial protein-coding genes was also seen in the woodlouse (*Armadillidium vulgare*) parasite *Thaumamermis cosgrovei* [121], suggesting that a low mutation rate in mitochondrial protein-coding genes may be a common feature of *Thaumamermis* spp.

RAPD (see Table 1) analysis of *Blatticola blattae*, a parasite of cockroaches (*Blattella germanica*), showed that the parasite population genetic structure closely mirrored that of the host, with both showing differentiation among buildings within cities, and among cities 900 km apart [122]. This strong genetic structuring likely reflects limited dispersal in cockroaches, promoting low gene flow in both parasite and host. Unlike *H. marelatus*, *B. blattae* is not markedly pathogenic and individuals form long-term associations with their hosts [123], so that there is time for host movement to mediate parasite gene flow.

Among parasitic nematodes of arthropods, then, pathogenicity to the host may influence the parasites’ population genetic structure, as parasites that kill their hosts very quickly cannot rely on host movement for dispersal and gene flow. However, where the arthropod host has a very small home range and does not disperse far, even largely non-pathogenic parasites may have strongly structured populations, as seen in *B. blattae* [122]. Nevertheless, our knowledge of the population genetics of parasitic nematodes of arthropods is very incomplete, and future work analysing a broader range of both host and parasite life histories is needed if we are to better understand the factors influencing their population genetics.

## Influence of anthropogenic disruption on parasitic nematode population genetics

Human activities have affected the geographical ranges of many host species, either shrinking a range through habitat loss, or increasing it through introduction of individuals into new regions. In many cases, the timing and extent of range changes are well documented, offering an opportunity to study how changes in host population size and connectivity shape parasitic nematode population genetics.

*Baylisascaris schroederi* is a nematode thought to be specific to giant pandas (*Ailuropoda melanoleuca*). Sequence analysis of both ITS and mtDNA have shown a lack of genetic differentiation among *B. schroederi* from geographically isolated panda populations [124–127], which is surprising because pandas do not migrate among populations, and pandas from each of these populations are themselves genetically differentiated [128]. The likely explanation for this difference between host and parasite population genetics is that *B. schroederi* has a much larger Ne than its host [129] and so undergoes population differentiation more slowly. Thus, while there may have been time for panda populations to differentiate through genetic drift in the 200 years since habitat fragmentation began [130], drift may not have been fast enough to yet differentiate *B. schroederi* populations. It has also been suggested that *B. schroederi* gene flow may occur among panda populations in the absence of panda movement, for example through association with a presently unknown paratenic host [127].

An analogous situation is seen in *Trypanoxyuris minutus* and *T. atelis*, parasites of the primates *Alouatta* spp. and *Ateles geoffroyi*, respectively, in Mexico. Since 1940, on-going forest fragmentation has created discontiguous host populations, among which host migration is very rare [131]. Despite this, mitochondrial sequence analysis of *Trypanoxyuris* spp. showed that parasite populations in different forest fragments were not genetically differentiated [132]. There are two non-mutually-exclusive explanations for this; unexpectedly high *Trypanoxyuris* gene flow among forest fragments, and/or the failure of *Trypanoxyuris* populations to detectably differentiate since becoming reproductively isolated. The latter explanation assumes that *Trypanoxyuris* populations were genetically unstructured prior to forest fragmentation, and this seems plausible as parasitic nematodes from a range of wild primates show limited population genetic structure when looking within host species [133–139].

The population genetics of *Baylisascaris procyonis* infecting raccoons (*Procyon lotor*) has been studied using both ribosomal and microsatellite loci in its native range [140, 141], with microsatellite loci revealing genetic differentiation across the Grand River (Michigan, USA). Among invasive *B. procyonis* populations in Germany, two well-differentiated clades have been detected by both ITS and mitochondrial sequence analysis, suggesting two independent introductions of *B. procyonis* into Germany [142]. Both German *B. procyonis* clades showed very low genetic diversity, likely the result of population bottlenecks (see Table 1) in the founding populations [142]. Low genetic diversity was seen also in *Rhabdias pseudosphaerocephala* in invasive cane toads (*Bufo marinus*) in Australia [143], and in *Passalurus ambiguus* in invasive rabbits (*Oryctolagus cuniculus*) in China [144], and is typical due to founder effects in these introduced species [145].

The nematode *Spirocamallanus istiblenni* was introduced to the Hawaiian archipelago with one of its hosts, the bluestripe snapper (*Lutjanus kasmira*). Population genetic analysis of *S. istiblenni* confirmed that the parasite originated from French Polynesia and showed that the introduced population was less genetically diverse than the native population [146]. Population genetic data also provided evidence that the introduced *S. istiblenni* has transmitted to native fish, shown by a lack of genetic differentiation between parasites from introduced and native hosts. Population genetic investigations into *Camallanus cotti*, invasive in Hawaiian stream fishes, revealed its probable invasion history, suggesting an initial introduction in O’ahu, where genetic diversity was highest, and subsequent migration to other islands in the archipelago [147]. Comparisons of the data in this study with data from *C. cotti* in its native range [115] showed that genetic diversity in introduced *C. cotti* was reduced compared with native populations, once again demonstrating the effects of population bottlenecks in introduced parasitic nematode populations.

*Anguillicoloides crassus* and its host, the Japanese eel (*Anguilla japonica*), were introduced from Asia to North America and Europe, and since then *A. crassus* has spread rapidly in European and American eels (*Anguilla anguilla* and *A. rostrata*, respectively), causing severe pathology in these naïve host species [148]. Population genetic studies have revealed multiple, distinct lineages of invasive *A. crassus*, suggesting multiple introduction events from different source populations [149, 150]. Furthermore, a southern to northern clinal decrease in its genetic diversity is seen in Europe, suggesting that *A. crassus* was introduced in southern Europe and has since spread northwards [150]. Hence, the population genetics of *A. crassus* has revealed its introduction history. Infrapopulation differentiation in *A. crassus* was studied in two European rivers, one of which was regularly artificially restocked with eels from a variety of sources and one in which eels had arrived by natural dispersal [151]. This showed that in the restocked river, *A. crassus* had high genetic diversity among hosts and a substantial deviation from Hardy-Weinberg equilibrium (see Table 1), while in the river with natural recruitment, there was no among-host structuring or deviation from Hardy-Weinberg equilibrium. These contrasting patterns were thought to be because the introduced eels had retained *A. crassus* infrapopulations reflective of their genetically distinct source populations, while *A. crassus* in the river with natural recruitment are derived from a single population that was already at Hardy-Weinberg equilibrium [151].

As environments continue to change, the ranges of parasitic nematodes will change too. The population genetics of parasitic nematodes currently undergoing such range changes show that (i) invasive parasitic nematodes are likely to have low genetic diversity (due to population bottlenecks); and (ii) that host populations are likely to lose diversity more rapidly following habitat fragmentation than parasitic nematode populations (due to the comparatively smaller Ne of hosts). This latter effect may have a consequence for parasite ecology, since hosts will differ in their genetic resistance to parasites, and genetic bottlenecks of host populations may therefore lead to altered degrees of parasitism. Supporting this, *Trypanoxyuris* spp. and *B. schroederi* all show genetic evidence of recent population expansion despite marked declines in host population size [127, 132], suggesting that prevalence and/or intensity of infection has increased since habitat fragmentation. Hence, population genetic analysis can inform on the biology of parasitic nematodes undergoing changes in range, and can be used to make predictions about how parasite populations might respond genetically to future range changes.

## Prospects in nematode population genetics

### Genome sequencing

Each of the methods routinely used to analyse parasitic nematode population genetics detects variation in a small number of loci [24], and it is often not clear how representative these loci are of a genome more widely. High-throughput sequencing techniques can be employed to interrogate large portions of the genome, thus reducing the effect of bias at any one genomic region. Other advantages include the ability to screen the genome for regions under selection, and the chance to analyse population genetic structure at several scales simultaneously. For example, highly polymorphic (see Table 1) genomic regions can inform on structure at very local scales, while more conserved regions will be appropriate for studying the relationships of more divergent populations. Genome-wide sequencing has been used to assess population genetics in non-nematode helminths, allowing detailed insight into parasite population genetic structure [152], and there is no reason why such insights should not be possible in parasitic nematodes. These techniques usually require a reference genome, which often will not be available *a priori* for non-model parasitic nematodes infecting wild animals. However, rapid advances in the sequencing and assembly of nematode genomes mean that it may often be feasible to generate a reference genome for the species in question [153].

In restriction-associated DNA sequencing (RADSeq) the genome of an individual is digested with a restriction endonuclease, and the resulting fragments are size-filtered and then sequenced [154]. Random distribution of restriction endonuclease sites across the genome ensures that the sequenced fragments are representative of the whole genome. Double-digest RADSeq (ddRADSeq) is a related technique that uses two restriction endonucleases [155]. While (dd)RADSeq data can be used without a reference genome assembly [156], having a reference means that non-target DNA can be detected and excluded. (dd)RADSeq approaches have not yet been used in the population genetic analysis of parasitic nematodes, but have been used in several other animal species [9, 157] including the free-living nematode *Caenorhabditis elegans* [158]. The study in *C. elegans* not only revealed the population genetics of this species, but also its recent evolutionary history, finding that the low genetic diversity of *C. elegans* likely arose from recent selective sweeps (see Table 1), in which a few beneficial alleles drove large swathes of the genome to near-fixation (see Table 1) due to extensive linkage disequilibrium (see Table 1) [158]. If (dd)RADSeq were used to study the population genetics of parasitic nematodes, we could expect similar insights into the biology and evolutionary history of these species.

In whole-genome sequencing, individuals are genotyped at virtually every locus polymorphic among samples. This allows the relationships among individuals, and hence the genetic structure of populations, to be resolved at the finest possible scale [159], and gives more power to make inferences about the evolutionary processes acting on a species [160, 161]. There are currently obstacles to routinely generating whole-genome sequence data. Firstly, sequencing the genomes of multiple individuals is often still expensive, such that there is a trade-off between the number of individuals sequenced and the genome coverage of each individual. However, population genetic techniques are robust to low genome coverage, with coverage of as little as two-fold having been applied [162]. Secondly, the small physical size of some nematodes can make it difficult to extract sufficient DNA for sequencing. Whole-genome amplification (WGA) prior to sequencing can ameliorate this difficulty [163], though not without some bias in genome coverage [164].

However, the difficulties of whole-genome sequencing are worth overcoming, due to the wealth of information it can provide. Of particular importance is the ability of whole-genome sequencing to identify genes that are under selection, and in the case of parasites, these genes may be relevant to pathogenicity, epidemiology and control. For example, whole-genome sequencing of the malaria parasite *Plasmodium falciparum* has found that genes that function in evasion of host immunity and in resistance to drugs show signs of selection [165]. Similar studies in parasitic nematodes are warranted, as anthelmintic resistance is an increasingly serious problem in both agricultural and medical settings, and the biochemical mechanisms of resistance are often poorly understood [20, 166]. Genes that show strong signatures of directional selection in anthelminthic-exposed populations are good candidates for genes conferring anthelmintic resistance [167].

Whole-genome sequencing has already been used to study the population genetics of parasitic nematodes that infect people, demonstrating among-host differentiation in *Wuchereria bancrofti* [168], and detecting differentiation in *Strongyloides stercoralis* among host individuals and populations [169]. However, these studies used small numbers of hosts and are limited in scope. Hence, there is now a need for studies on a comparatively larger scale that examine the distribution of polymorphisms within genomes as well as among them. In an intriguing recent study, whole-genome sequencing was used to interrogate the population genetics of the plant-parasitic nematode *Heterodera glycines* without the use of a reference genome [170]. This study, which made use of the UNEAK bioinformatics pipeline [171], was able to not only elucidate the population genetic structure of *H. glycines*, but also to identify genetic variants showing signatures of selection. Whether a reference assembly is used or not, whole-genome sequencing studies will improve our understanding of how parasitic nematodes respond to natural selection pressures. As the climate changes, parasitic nematodes will encounter novel selection pressures, and how they respond to these pressures may have important consequences, not only for host species but for the ecosystem as a whole.

### Environmental DNA (eDNA) analysis

Parasitic nematodes typically have extra-host transmission stages in their life-cycle, and as these stages are the pool from which the next generation of parasites will arise, they contribute directly to Ne. Differences in the number, spatial distribution and temporal distribution of these stages may therefore influence the rate of genetic drift in a population, and hence the population genetic structure more widely. Despite their likely importance in both population genetics and epidemiology, very little is known about the ecology of extra-host stages, and what is known largely pertains to human or livestock pathogens [172, 173]. This knowledge gap is principally due to the difficulty in sampling and identifying extra-host nematode stages in the environment. Sequencing of environmental DNA (eDNA, see Table 1) may be a solution to this problem [174, 175]. DNA-based identification of parasitic nematodes stages in host faeces - in essence a form of eDNA - is often used to diagnose infection in livestock [176]. Adaptation of these techniques for the detection of parasitic nematode stages in soil and water (where target DNA concentrations will often be lower) would incorporate extra-host transmission stages into analysis of population genetics of parasitic nematodes infecting wild animals.

## Synthesis and outstanding research questions

As with all species, the population genetics of parasitic nematodes in wild animals is ultimately determined by the (i) rate of gene flow among populations and (ii) strength of genetic drift within populations. Nematodes have very limited dispersal ability of their own, so that they are largely dependent on their hosts for dispersal. Therefore, in principle, nematode populations should ultimately come to be structured at the scale over which hosts move. Broadly speaking, this rule is largely followed - there is very limited population genetic structuring of parasitic nematodes infecting highly mobile hosts, such as ocean-going mammals [100], while the parasites of small, sedentary rodents, for example, generally have highly structured populations [15].

Nematode species that use more than one host species often have less structured populations than the movement of any one host species would predict. This has been observed in *Trichinella* spp., which shows gene flow at the continental scale [38], in *Dictyocaulus eckerti*, a generalist parasite of deer [86], and in *Neoheligmonella granjoni* [72, 73]. The use of multiple mobile host species allows nematodes to traverse or colonise a wider range of habitats than would be possible using only a single host, and this tends to promote parasite gene flow. In contrast, highly pathogenic parasitic nematodes, such as *Heterorhabditis marelatus*, may show reduced gene flow if they significantly hamper their host’s movement [116].

In the absence of gene flow all populations will ultimately diverge genetically due to genetic drift, but this process takes time. How much time depends on the strength of genetic drift, in turn dictated by (among other things) Ne, and so Ne is a major determinant in population genetic structure. Ne is rarely measured directly but can be estimated from known aspects of parasite and host life history. For example, haploid males in *Syphacia stroma* likely reduce Ne in comparison with the fully diploid *Heligmosomoides polygyrus*, with which *S. stroma* shares a host [78], while the much lower infection prevalence and intensity of *Dictyocaulus viviparus* compared with *Ostertagia ostertagi* in domestic cattle means that the former likely has lower Ne [177, 178]. In both cases, the species presumed to have the lower Ne has stronger population genetic structuring. Naturally, parasites with a fast generation time will undergo more rapid genetic drift (and so faster population divergence) than parasites with a slow generation time. Indeed, the significantly faster generation time of *H. polygyrus* compared with *Trichuris muris* may contribute to the comparatively more strongly genetically structured populations of the former when both species are analysed from the same host individuals [15, 53, 54].

Parasite life history traits such as host range, reproductive strategy, generation time, and the prevalence, intensity and pathogenicity of infection in the host are therefore all important in determining the population genetics of parasitic nematodes. These factors interact with host ecology, and in particular, host movement behaviour, to establish patterns of parasite population genetic structure. However, the relative importance of parasite life history traits remains poorly understood. Animals are commonly infected by more than one species of nematode, and this fact could be exploited to better understand population genetics in nematodes infecting wild animals. For example, comparison of life history traits among co-infecting parasites would allow their effects on parasite populations genetics to be separated from host-dependent effects.

The abundance and spatial and temporal dynamics of extra-host stage parasitic nematodes in the environment remains almost entirely unknown. There have been some attempts to study the extra-host stages of nematodes infecting domestic livestock [179], but the findings may not be fully applicable to species infecting wild animals. Understanding the ecology of extra-host parasite stages is important for conservation of wild hosts, for monitoring the threat of zoonotic infection, and for our understanding of ecosystem processes. Particularly mysterious is the role of extra-host stages on the population genetics of parasitic nematodes, and future work must address this to complete our understanding of the population genetics of parasitic nematodes in wildlife. eDNA applications may be the only means of sampling extra-host nematode stages with sufficient rigour to understand their contribution to population genetics.

Looking to the future, the use of high-throughput sequencing-based methods (dd)RADSeq and whole-genome sequencing) will dramatically improve the resolution and accuracy with which population structure can be detected. Furthermore, whole-genome sequencing will allow other aspects of parasitic nematode genomes, such as the size and nature of selection, and the extent of linkage disequilibrium, to be interrogated, and this knowledge will improve our understanding of parasitic nematode biology.

## Conclusions

The population genetics of parasitic nematodes in wild animals is determined by a combination of host ecology - especially host movement behaviour - and parasite life history. Studying the population genetics of parasitic nematodes of wild animals can reveal how their populations respond to selective pressures. With this information, we can assess the risk parasitic nematodes pose to natural ecosystems and to the health of humans and domestic animals, as anthropogenic activities drive environmental changes and changes in species’ geographical ranges. Our understanding of the population genetics, biology and evolutionary history of parasitic nematodes will be improved if investigators incorporate extra-host transmission stages and take advantage of high-throughput DNA-sequencing technologies in future studies.

## Abbreviations

ddRADSeq: Double-digest restriction-associated DNA sequencing
DNA: Deoxyribonucleic acid
eDNA: Environmental DNA
ITS: Internal transcribed spacer
mtDNA: Mitochondrial DNA
N: Census size
Ne: Effective population size
PCR: Polymerase chain reaction
RADSeq: Restriction-associated DNA sequencing
RAPD: Random amplified polymorphic DNA
