# Correction to: The population genetics of parasitic nematodes of wild animals

**DOI:** 10.1186/s13071-019-3731-1

**Published:** 2019-10-09

**Authors:** Rebecca Cole, Mark Viney

**Affiliations:** School of Biological Sciences, University of Bristol, Bristol, BS8 1TQ UK

## Correction to: Parasit Vectors (2018) 11:590 10.1186/s13071-018-3137-5

Unfortunately, the original version of this article [1] contains an error. In the section entitled “Influence of anthropogenic disruption on parasitic nematode population genetics”, the passage

- “Population genetic studies have revealed multiple, distinct lineages of invasive *A. crassus*, suggesting multiple introduction events from different source populations [149, 150]. Furthermore, a southern to northern clinal decrease in its genetic diversity is seen in Europe, suggesting that *A. crassus* was introduced in southern Europe and has since spread northwards [150].”

should read

- “Population genetic studies have revealed multiple, distinct lineages of invasive *A. crassus* in North America *versus* Europe, suggesting introductions from different source populations [149, 150]. Furthermore, a northern to southern clinal decrease in its genetic diversity is seen in Europe, suggesting that *A. crassus* was introduced in northern Europe and has since spread southwards [150].”

The authors apologize for the inconvenience caused.
